# Insights from neuroradiology in acute disseminated encephalomyelitis

**DOI:** 10.1016/j.radcr.2023.09.020

**Published:** 2023-09-26

**Authors:** Shivam Khatri, Michael Xerras, Salomon Chamay, Shorabh Sharma

**Affiliations:** a CUNY School of Medicine, New York, NY 10031, USA; b Division of Internal Medicine, Department of Medicine, St. Barnabas Hospital, Bronx, NY 10457, USA

## Abstract

Acute disseminated encephalomyelitis (ADEM) is a rare immune-mediated demyelinating disorder primarily observed in children, typically triggered by recent vaccination or viral infection. Although uncommon, there have been reports of ADEM occurring in adults, with varying radiological findings compared to pediatric cases. Distinguishing ADEM from other demyelinating disorders, such as multiple sclerosis (MS) and neuromyelitis optica (NMO), can pose a diagnostic challenge. We present a case report of an 18-year-old male with a history of polysubstance use who was successfully treated with corticosteroids and highlight the neuroradiological presentation of ADEM, emphasizing the importance of accurate diagnosis and management in both pediatric and adult populations.

## Introduction

Acute disseminated encephalomyelitis (ADEM) is a central nervous system disorder primarily observed in children that is characterized by multifocal symptoms [1,2]. It frequently emerges after a viral infection or recent vaccination [2]. ADEM manifests with symptoms such as fever, vomiting, headaches, and altered mental status [3]. Additionally, clinical features may include visual field defects, aphasia, motor and sensory deficits, ataxia, and movement disorders [4]. Typically, ADEM follows a monophasic clinical course, and instances of relapsing ADEM are rare [5]. Due to a limited understanding of the disease, ADEM is sometimes mistaken for multiple sclerosis (MS) or other demyelinating diseases [2,6].

The annual incidence of ADEM is reported to be approximately 0.4-0.8 per 100,000 individuals, with the highest occurrence among young children (mean age of approximately 6-8 years), particularly during the winter and spring months [4]. ADEM cases in adults are infrequent, and the clinical course within this age group remains mostly unknown [7]. Although 50%-75% of adult cases recover, around 20% progress to multiple sclerosis [6]. In this report, we present the case of an 18-year-old who developed ADEM and experienced recovery after receiving a trial of IV methylprednisolone. Our aim is to discuss the various neuroradiological findings that can present in ADEM.

## Case presentation

An 18-year-old male with a history of polysubstance abuse, specifically oxycodone, presented to the emergency room (ER) with complaints of left-sided weakness persisting for 1 week. The onset of weakness followed an incident where he attempted to jump over a gate but fell instead. Despite being able to stand and walk after the fall, he experienced weakness throughout the left side of his body. On the same day, he engaged in marijuana use and consumed oxycodone 15 mg. Upon waking up the following day, he noticed a worsening of the left-sided weakness. He reported a tendency to walk predominantly relying on his right side, experiencing slurred speech, and observing reduced sensation to touch on the left side of his body. He came to the ER 3 days prior to this, but he left against medical advice (AMA) because he did not want to wait for an MRI. He had no recent history of viral illness, sick contacts, or vaccinations.

Upon admission to the ER, the patient's vital signs were recorded as follows: body mass index of 18.4, temperature of 98.0°F, blood pressure of 138/93 mm Hg, mean blood pressure of 108, heart rate of 80 beats per minute, and respiratory rate of 19 breaths per minute. Physical examination was notable for facial asymmetry on the left side with an asymmetrical nasolabial fold. Motor strength of the left lower extremity was 3/5 and his gait was ataxic with a tendency to ambulate towards his right side. In comparison, right upper and lower extremity strength was 5/5. The cranial nerve examination yielded unremarkable results with the exception of facial asymmetry, and no sensory deficits were identified. Cardiovascular, pulmonary, and abdominal examinations did not reveal any significant findings. Imaging studies conducted in the emergency room included noncontrast magnetic resonance imaging (MRI) and magnetic resonance angiography (MRA) of the brain, as well as noncontrast computed tomography (CT) scans of the brain and cervical spine.

**Fig. 1:**
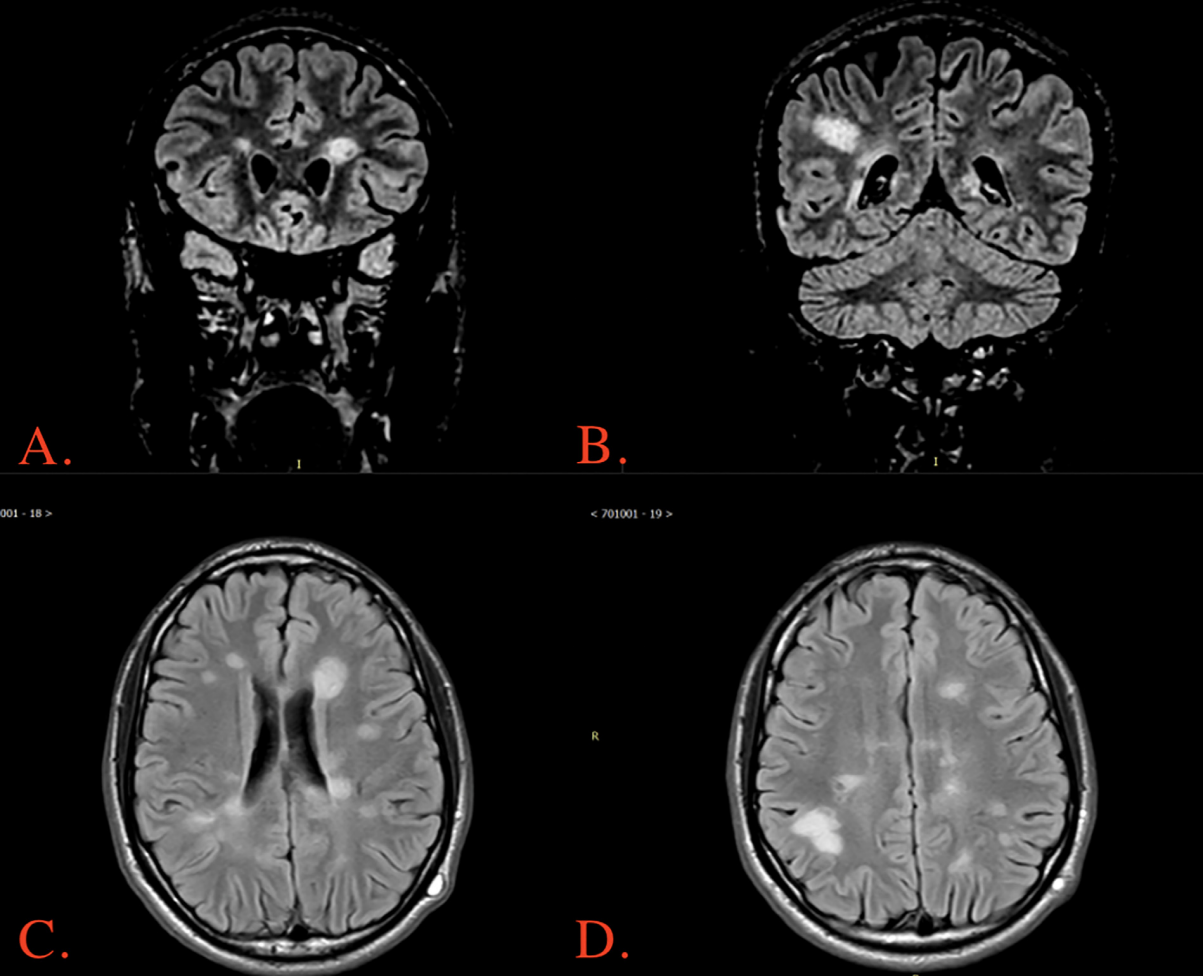
(A and B) Coronal FLAIR showing T2 signal intensity affecting both the supratentorial and infratentorial regions of the brain (C and D) Axial FLAIR showing T2 signal intensity affecting both the supratentorial and infratentorial regions of the brain.

The MRI brain revealed the presence of multiple areas with prolonged T2 signal intensity affecting both the supratentorial and infratentorial regions of the brain. The involvement was primarily observed in the deep white matter, with a few scattered foci affecting the subcortical gray matter and the right thalamus (Fig. 1).

Based on the assessment of the radiologist, these findings were consistent with acute demyelinating encephalomyelitis (ADEM).

**Fig. 2:**
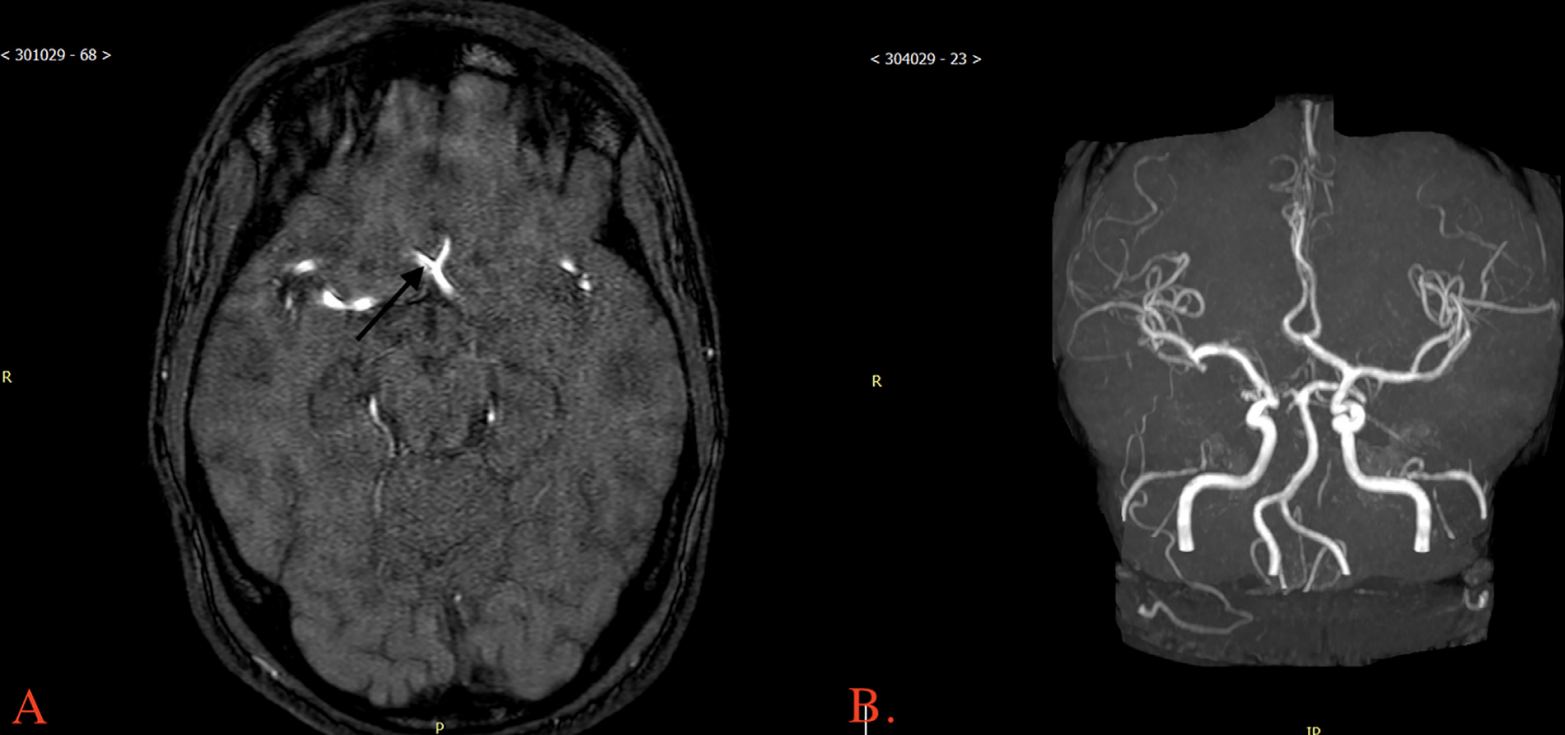
(A) MRA brain with arrow pointing to hypoplastic right A1 segment. (B) 3D reconstruction of MRA brain showing hypoplastic right A1 segment.

The MRA brain showed a hypoplastic right A1 segment (Fig. 2).

**Fig. 3:**
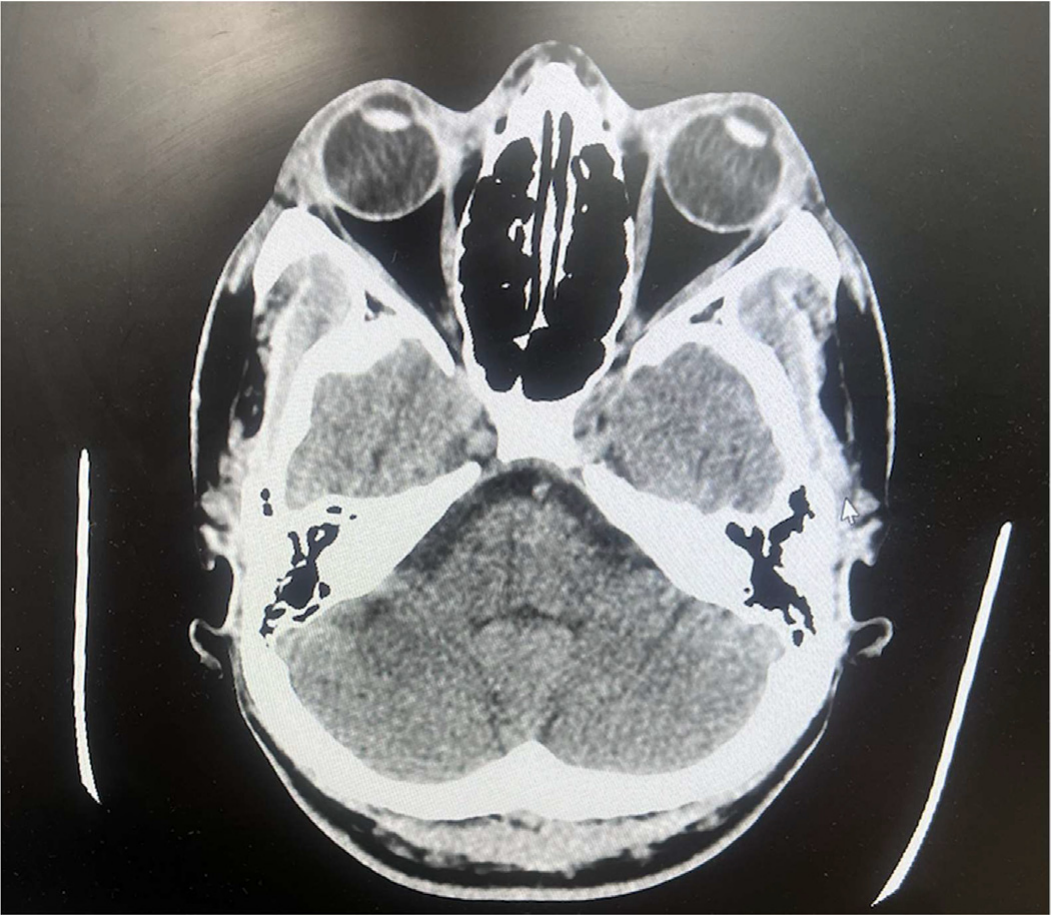
Axial view of CT brain without contrast showing no abnormal findings.

The CT Brain without contrast (Fig. 3) and CT cervical spine without contrast showed no acute intracranial abnormalities and no vertebral abnormalities.

In the ER, the patient underwent several laboratory studies, including a complete blood count, basic metabolic panel, lipid profile, hemoglobin A1C, antinuclear antibodies, erythrocyte sedimentation rate, and hepatitis C testing on polymerase chain reaction. The results of these tests indicated that all parameters were within the reference range.

The patient was admitted to the hospital floor and evaluated by the neurology team. During the neurological examination, the patient exhibited dysarthria, a slight pronator drift in the left upper extremity, and weaker grip strength on the left side. After consulting with neuroradiology, the neurology team suspected ADEM and recommended further investigations. They suggested conducting a contrast-enhanced MRI of the brain, HIV testing, serologic testing, and a lumbar puncture. They also mentioned that treatment with high-dose steroids would be considered once the serum test results were available.

On day 1 of admission, MRI brain and MRA brain without and then with contrast showed similar findings to those performed in the ER. A lumbar puncture demonstrated a clear, colorless appearance with elevated white blood cells at 26 mm^3^, elevated lymphocytes at 92%, and a low monocyte count at 8%. Cerebrospinal fluid cultures were negative. Testing for oligoclonal banding, antinuclear antibodies, antidouble stranded DNA antibodies, Hepatitis C antibodies, VDRL, Sjogren's antibodies, antismith antibodies, and anti-RNP (ribonucleoprotein) antibodies were negative. Per recommendations of neurology, the patient was started on IV methylprednisolone 1000 mg every day for 3-5 days. On day 2 of admission, ophthalmology was consulted to rule out prior optic neuritis, and examination indicated no signs of optic disc pathology. On day 3 of admission, the patient reported feeling better since beginning the steroid course. By day 4, the patient continued to report improvement of symptoms, including left-sided weakness. No deficits in motor strength were present at this point and the patient was discharged on day 5 with instructions to switch to oral prednisone for 6 weeks in a tapering dose regimen. Follow-up appointments were scheduled with an outpatient primary care provider and neurologist, and the patient was seen at follow-up appointments. Strength remained intact. He came back to the emergency room 30 days later (⅘) complaining of 5-6 episodes of muscle spasms per day. Each episode lasted for a few minutes at a time, and he complained of intermittent episodes of rigidity in his muscles. He was seen by a neurologist, who recommended admission for an MRI brain and further workup to make sure the disease process was not worsening. However, he left AMA and was lost to follow up.

## Discussion

ADEM typically occurs in pediatric patients and is considered rare in adults. A study conducted in China found that the incidence of ADEM decreases with age, particularly among individuals older than 50 years [8]. Within the adult age group, incidence is highest among those aged 50-59 years, followed by the 60-69 age group [8]. The case being discussed presents a unique scenario, as it involves an 18-year-old male without a definitive known trigger who was diagnosed with ADEM. Exploring the underlying mechanism and understanding the neuroradiological presentation of ADEM in various age groups could potentially provide valuable insights into this disease.

ADEM is often linked to postviral or postvaccination occurrences; however, it can also manifest without a clear connection to recent infections or vaccinations [2]. While the prevailing "molecular mimicry" hypothesis suggests that autoimmune reactions to myelin may arise in genetically susceptible individuals, an alternative hypothesis posits that an inflammatory response to a foreign antigen could trigger increased permeability and vascular congestion within the central nervous system [2]. Consequently, this vascular disruption may lead to demyelination, gliosis, and necrosis. In the case of this patient, it is plausible to consider the fall as a potential triggering event for their ADEM, although further investigation is necessary to establish a definitive cause. Perhaps the use of substances such as Oxycodone may have contributed to a triggering event for ADEM.

To better understand the diagnostic features that led neurology and neuroradiology to suggest a diagnosis of ADEM in this patient, it is crucial to comprehend the radiological characteristics associated with ADEM. ADEM typically manifests as multiple T2 hyperintense white matter lesions that can affect both hemispheres [2]. These lesions are commonly located near the cortical region and involve U-fibers. Furthermore, ADEM often exhibits symmetric gray matter lesions on fluid-attenuated inversion recovery (FLAIR) images, primarily observed in the basal ganglia and thalamus [2]. In about one-third of cases, the spinal cord may also be affected. These radiological findings aid in distinguishing ADEM from other demyelinating disorders like MS and NMO [2]. When comparing ADEM with MS on MRI, MS lesions typically exhibit better demarcation compared to the less distinct lesions found in ADEM. Additionally, T1-weighted MRI scans with findings such as the black hole sign are more indicative of MS [6]. MS lesions tend to appear in both the subcortical white matter and the periventricular margin [9]. On the other hand, MRI scans of ADEM show patchy, increased signal intensity on conventional T2-weighted imaging and FLAIR MRI [6]. ADEM lesions are predominantly located in the subcortical white matter, with relatively spared periventricular white matter [9].

In adult patients with ADEM, midbrain lesions on MRI are more commonly observed compared to lesions in the pons or medulla oblongata [10]. In pediatric patients with ADEM, brainstem lesions tend to involve the pons more frequently. Hence, current data suggests that the location of brainstem lesions differs between adult and pediatric patients with ADEM [10].

Although rare, there are other documented case reports in the literature involving adults presenting with acute Disseminated Encephalomyelitis. Almaghrabi et al. [2] reported a case of a 40-year-old male who presented to the emergency department with decreased consciousness, delirium, and eventually tonic-clonic convulsions that were alleviated with diazepam. This patient had no recent history of infections or vaccinations, and the MRI revealed multiple high T2 abnormalities in the white matter [2]. There was no follow-up for the patient in that case report. Our case shares similarities, as our patient also lacks recent viral infections or vaccinations and exhibits multiple high T2 abnormalities. In our case, the patient exhibited T2 signal intensities in the supratentorial and infratentorial regions of the brain, whereas the patient described in the study by Almaghrabi et al. [2] presented signal intensities in the white matter of the supratentorial cortical and subcortical parieto-occipital region.

In a case described by Liardino et al. [6], a 47-year-old male presented to the emergency department with bilateral lower extremity weakness and a spastic gait. He had experienced diffuse abdominal cramping pain four days prior. The initial MRI with contrast enhancement demonstrated multiple T2-weighted, contrast-enhancing, diffuse patchy lesions in both cerebral hemispheres [6]. The patient received intravenous methylprednisolone 500 mg twice daily for 5 days and showed a similar positive response as our patient, both in terms of radiological findings and the successful outcome following the 5-day course of IV methylprednisolone treatment.

The primary treatment approach for ADEM is the administration of high-dose intravenous (IV) steroids, typically methylprednisolone [7]. The therapeutic dosage ranges from 10-30 mg/kg/d for a duration of 3-5 days [8]. Patients receiving methylprednisolone often experience substantial improvement in their neurological symptoms. While dexamethasone can also be utilized, methylprednisolone has demonstrated superior outcomes [8]. In cases where ADEM does not respond adequately to initial therapy, advanced treatments like Intravenous Immunoglobulin and plasmapheresis may be considered [7]. It is important to note that vaccination should be avoided for at least 6 months following recovery [8].

Other differentials to consider include vascular malformations of the central nervous system, including carotid atherosclerosis complicated by rupture of the plaque. Neovascularization of the plaque is considered to be a potential risk factor for carotid plaque rupture. Contrast-enhanced ultrasound (CEUS) may be used to detect neovascularization within carotid vessels [11].

## Conclusion

Though clinically uncommon, ADEM can present in adults and may oftentimes be difficult to differentiate from multiple sclerosis or other demyelinating disorders. Neuroradiologists play a critical role in examining these differences, such as multiple T2 hyperintense white matter lesions, which are characteristic of ADEMs. This case underscores the significance of multidisciplinary approaches and highlights the invaluable contributions of neuroradiologists and neurologists in guiding management of ADEM and enhancing our understanding of this neurological disease.

## Patient consent

Informed consent was obtained from the patient before writing up this case study.
